# Quantification of Ceftazidime in the Vitreous Humor Using Ultra-performance Convergence Chromatography–Tandem Mass Spectrometry

**DOI:** 10.1097/FTD.0000000000001371

**Published:** 2025-08-21

**Authors:** Soma Bahmany, Michele Manzulli, Boudewijn van der Wel, Koorosh Faridpooya, Saskia van Romunde, Rawi Ramautar, Robert B. Flint

**Affiliations:** * Department of Hospital Pharmacy, Erasmus MC, University Medical Center Rotterdam, Rotterdam, the Netherlands;; † Rotterdam Eye Hospital, Rotterdam, the Netherlands;; ‡ Leiden Academic Center for Drug Research, Leiden University, Leiden, the Netherlands; and; § Division of Neonatology, Department of Neonatal and Pediatric Intensive Care, Erasmus MC Sophia Children's Hospital, Rotterdam, the Netherlands.

**Keywords:** ceftazidime, minimal inhibitory concentration, vitreous humor, endophthalmitis, target-site concentration

## Abstract

**Background:**

Patients with suspected postoperative bacterial endophthalmitis are at a high risk of vision loss if not treated immediately and adequately. Initial treatment typically involves the intravitreal administration of antibiotics, with ceftazidime in combination with vancomycin being the common agents administered. Unbound ceftazidime exposure should exceed the minimum inhibitory concentration at the infection site. To facilitate investigation of the disposition of ceftazidime after its intravitreal injection, an ultra-performance convergence chromatography–tandem mass spectrometry method for quantifying ceftazidime in the vitreous humor was developed and validated in this study.

**Methods:**

Each sample (20 µL) was prepared by protein precipitation of the test sample mixed with the internal standard solution (2 mg/L meropenem-d_6_ in methanol). The sample was analyzed using a Waters Acquity UPC^2^ system coupled to a Waters Xevo TQ-S micro triple quadrupole mass spectrometer (Waters Corp, Milford, MA). The method was validated according to guidelines published by the European Medicines Agency and US Food and Drug Administration. The validation parameters were linearity, limits of quantification (LOQs), accuracy, interday and intraday precision, carryover effect, autosampler stability, and short-term and long-term stability.

**Results:**

The method had a linear range (*r*^2^ > 0.990) between 1.3 and 99.6 mg/L and exhibited less than 15% inaccuracy and imprecision. The carryover effect was significant (53% of the lower LOQ) when injecting a blank sample after an upper LOQ sample but was negated after injecting an additional blank sample. Therefore, 1 blank sample should be injected after each patient sample.

**Conclusions:**

This ultra-performance convergence chromatography–tandem mass spectrometry method facilitates the rapid and reliable determination of ceftazidime in the vitreous humor, with a short run time of 5 minutes. It was successfully applied to 72 clinical samples.

## INTRODUCTION

Patients with suspected postoperative bacterial endophthalmitis are at a high risk of rapidly losing their vision if not treated immediately and adequately. The initial treatment for patients with this inflammatory condition typically involves the intravitreal administration of antibiotics, with ceftazidime in combination with vancomycin being the most commonly administered drugs.^1^ Unbound ceftazidime exposure should exceed the minimum inhibitory concentration at the infection site. In such patients, the site of infection is the vitreous humor (VH), a homogeneous and viscoelastic hydrogel that is located between the lens and retina and occupies approximately 80% of the volume of an adult eye globe. Although the VH comprises mainly water (>90%), it also contains solid fibrillar components such as glycosaminoglycans and collagen and noncollagenous components such as glycoproteins and nonstructural proteins.^2^ However, these compositional properties can be affected by aging and diseases. Therefore, knowledge regarding the disposition of ceftazidime in patients with suspected postoperative bacterial endophthalmitis is required to guarantee adequate antibiotic treatment.

To investigate the exposure of ceftazidime after its intravitreal injection, a sensitive, accurate, and selective method for quantifying the drug in the VH is required. The authors have previously developed an ultra-performance convergence chromatography–tandem mass spectrometry (UPC^2^–MS/MS) method for analyzing beta-lactam antibiotics in various complex sample matrices.^3–5^ It would be of great interest to evaluate the potential of this UPC^2^ method for the reliable determination of ceftazidime in the VH. UPC,^2^ a chromatographic analytical separation technique based on supercritical fluid chromatography, is recommended for highly polar compounds such as beta-lactam antibiotics (including ceftazidime). The primary mobile phase (MP) consists of compressed carbon dioxide (CO_2_) combined with a small amount of an organic modifier. The use of CO_2_ as the primary MP component offers several advantages, as the gas is nontoxic, nonflammable, and relatively inexpensive.^6^ Moreover, the lower use of organic solvents in UPC^2^ compared with the amount used in liquid chromatography (LC) or hydrophilic interaction chromatography renders this method greener and more environmentally friendly, thereby adhering to the sustainable development goals of the United Nations. In previous studies, the authors successfully developed and validated UPC^2^–MS/MS-based methods for quantifying different beta-lactam antibiotics in plasma, synovial fluid, synovial tissue, and bone.^3–5^ Therefore, the aim of this study was to determine whether UPC^2^–MS/MS can achieve the same performance metrics in quantifying ceftazidime in the VH for clinical research purposes. Such an approach would enable investigations of the clearance of ceftazidime from the VH, which is required to determine the optimal administration dose for patients with suspected postoperative endophthalmitis. Method validation was performed according to international guidelines.^7,8^ To the best knowledge of the authors, this is the first report of the use of UPC^2^–MS/MS to quantify ceftazidime in the VH.

## MATERIALS AND METHODS

### Chemicals and Reagents

Ceftazidime was purchased from Sigma-Aldrich (St. Louis, MO) and Thermo Fisher Scientific (Waltham, MA) for the calibration standards and quality control (QC) samples, respectively. Meropenem-d_6_ was purchased from Santa Cruz Biotechnology Inc (Huissen, the Netherlands). Ammonium formate was purchased from Honeywell Research Chemicals (Landsmeer, the Netherlands). LC–MS-grade methanol (99%) and formic acid were obtained from Biosolve (Valkenswaard, the Netherlands). Milli-Q water was produced using a Milli-Q Advantage A10 water purification system (Merck Millipore, Darmstadt, Germany). Ceftazidime-free (blank) VH samples were obtained from the residues of patients during eye surgery at the Rotterdam Eye Hospital in the Netherlands.

### Stock Solutions, Internal Standard, Calibration Standards, and Quality Control Samples

#### Stock and Working Solutions

The calibration standards and QC samples were prepared from separate 1000 mg/L stock solutions that were prepared in Milli-Q water. The 100 mg/L stock solution of the internal standard (meropenem-d_6_) prepared in methanol was diluted to 2 mg/L as a working solution and stored in light-resistant containers at −20°C for up to 3 months.

### Calibration Standards and Quality Controls

The calibration curve included 8 standards, all of which were prepared by diluting the appropriate solution with the blank VH as follows: calibration standard 8 was prepared by diluting the stock solution; calibration standards 5, 6, and 7 were prepared by diluting standard 8; calibration standards 3 and 4 were prepared by diluting calibration standard 6; and calibration standards 1 and 2 were prepared by diluting calibration standard 3. The QC samples were prepared at 4 concentrations: lower limit of quantification (LLOQ), low (L), medium (M), and high (H). These were prepared by diluting the appropriate solution with the blank VH as follows: QC H and QC M were prepared by diluting the stock solution; QC L was prepared by diluting QC H; and QC LLOQ was prepared by diluting QC M. The concentrations of the calibration standards and QC samples are listed in Table 1. A 20 µL aliquot of each prepared solution was pipetted into 1.5-mL safe-lock Eppendorf tubes and stored at −80°C before analysis.

**TABLE 1.** Summary of the Concentrations of Calibration Standards and Quality Control Samples Including the Accuracy and Precision Results

Compound	Sample	Conc. (mg/L)			
Ceftazidime	S1	1.4			
	S2	2.9			
	S3	5.8			
	S4	11.5			
	S5	16.6			
	S6	23.0			
	S7	49.8			
	S8	99.6			
	Sample	Conc. (mg/L)	Accuracy (%)	Intraday precision (%)	Interday precision (%)
	LLOQ	1.3	0.3	5.4	9.1
	L	5.1	6.1	6.6	5.1
	M	33.3	3.2	1.4	3.9
	H	62.4	5.1	3.9	6.2

H, high; LLOQ, lower limit of quantification; L, low; M, medium; S, standard; QC, quality control.

#### Sample Preparation

First, 300 µL of the internal standard solution was added to 20 µL of the sample and mixed for 10 seconds. The mixture was then centrifuged at 1811*g* for 5 minutes. After centrifugation, 50 µL of the supernatant was pipetted into an amber 2-mL autosampler vial and diluted with 950 µL of eluent B (5% (v/v) 0.01 mol/L ammonium formate in methanol). The sample was mixed for 10 seconds, after which 20 µL was injected into the UPC^2^–MS/MS system.

### Instrumentation

The analysis was performed using a Waters Acquity UPC^2^–MS/MS system coupled to a Waters Xevo TQ-S micro triple quadrupole mass spectrometer (Waters Corp., Milford, MA). The Acquity UPC^2^ system consisted of a binary solvent manager, an isocratic solvent manager, a convergence manager, a sample manager, and a column manager. Data were processed using Masslynx 4.1 and Targetlynx 4.1 software (Waters Corp).

### Chromatographic Conditions

For chromatographic separation of the antibiotic and internal standard, a gradient elution program with 2 different MPs (A and B) was applied. MP A consisted of liquid CO_2_, whereas MP B consisted of 5% (v/v) 0.01 mol/L ammonium formate in methanol. The column temperature was set at 55°C and the flow rate at 0.75 mL/min. The gradient conditions were as follows: first, 70% MP A and 30% MP B for 1 minute; then MP A was decreased to 45% and maintained at this level for 3 minutes; and finally, MP A was restituted to 70% within 0.5 minutes to equilibrate at starting conditions. The total run time was 5.0 minutes, with the autosampler temperature set at 5°C.

### MS/MS Conditions

MS/MS detection was performed in the positive mode using electrospray ionization. The capillary voltage was 3.50 kV, the source temperature was 150°C, the desolvation temperature was 350°C, and the desolvation gas flow rate was 650 L/h. Because of atmospheric pressure, MP A (CO_2_) returns to the gas phase in the tubing that is linked to the mass spectrometer. Therefore, a separate MP B flow rate from the isocratic solvent manager was applied to the mass spectrometer to stabilize and optimize the ionization process. The optimized MS/MS settings for all compounds are summarized in Table 2.

**TABLE 2.** MS/MS Settings of Each Compound and the Internal Standard

Compound	ESI Mode	Parent Mass (m/z)	Daughter Mass (m/z)	Cone Voltage (V)	Collision Energy (V)	Dwell Time (ms)
Ceftazidime	+	546.97	468.03	22	10	50
Meropenem-d6	+	390.02	147.09	12	32	50

### Method Validation

Validation of the proposed analytical method was performed according to guidelines published by the European Medicines Agency (EMA) and US Food and Drug Administration (FDA).^7,9^ However, stricter requirements were used for linearity; that is, whereas the EMA and FDA guidelines state that at least 75% of the calibration standards should meet the acceptance criteria, this requirement was extended to all the calibration standards in this study. The following parameters were validated: linearity, limits of quantification (LOQs), accuracy, precision, carryover effect, stability, and matrix effects.

### Linearity

The blank samples (with or without internal standards) and 8 calibration standards were prepared and measured in duplicate to validate the linearity of the method. The back-calculated concentrations of the calibration standards should be within 15% of the nominal concentrations. The highest calibration standard was set as the upper LOQ (ULOQ). The correlation coefficient should be at least 0.995 and the determination coefficient was expected to be at least 0.990.

### Accuracy

All QC samples were measured 5 times on 3 different days. The biases of QC L, QC M, and QC H should be within 15% and that of QC LLOQ was expected to be within 20%.

### Intraday and Interday Precision

The intraday precision was assessed by measuring all QC samples 5 times on the same day. The interday precision was assessed by measuring all QC samples 5 times on 3 different days. All coefficients of variation should be within 15%, except for that of the LLOQ, which was expected to be within 20%.

### Stability

The stability of the autosampler was investigated by storing extracts of the prepared QC samples (L, M, and H) in duplicate in the autosampler at 5°C. The extracts were then measured against freshly prepared calibration standards after 24 and 72 hours of storage. Short-term stability was assessed by storing QC L and QC H samples in duplicate in the freezer at −20°C. These QC samples were then measured against freshly prepared calibration standards after 168 hours of storage. To assess long-term stability, QC L and QC H samples were stored in a −80°C freezer for up to 3 months, after which they were measured in duplicate against freshly prepared calibration standards. All measured concentrations of the QC samples were compared with the measured concentrations at Timepoint (T) = 0. The QC samples were considered to be stable if their recoveries were within 85%–115%.

### Carryover Effect

The carryover effect was assessed by analyzing 2 blank samples after analysis of the highest calibration standard. The measured concentrations in the blank samples should not exceed 20% of the LLOQ concentration.

### Matrix Effects

Matrix effects were assessed by comparing the slopes of the calibration curves generated from analyses of the compounds in different matrices.^10^ In total, 3 calibration curves, including 8 calibration standards, were prepared after the analysis of 3 different matrices: Milli-Q water, blank VH from pigs, and blank VH from human patients.

### Clinical Application to Patient Samples

In the conventional treatment of patients with suspected postoperative endophthalmitis, antibiotic administration is delayed until a vitreous biopsy is obtained because the growth of cultures depends on the presence of viable bacteria. Reducing the delay in antibiotic treatment is expected to improve patient outcomes. The authors were previously able to reverse the traditional order of treatment steps by using polymerase chain reaction-based diagnostics, which do not require viable bacteria; that is, antibiotics were administered immediately, followed by a planned biopsy.

According to the new treatment protocol implemented at the OZR-Rotterdam Eye Hospital since 2022, ceftazidime and vancomycin are administered immediately upon symptom presentation, saving valuable time. A vitreous biopsy is then scheduled for the same or following day for pathogen identification. The biopsy specimen is consistently collected from the same location in the eye; that is, 4 mm from the limbus in the temporal quadrant. The tip of the vitrectomy needle must be positioned centrally in the vitreous space to ensure optimal sampling of the vitreous body. After obtaining informed consent from patients who had undergone this new treatment protocol, 72 residual samples were collected for UPC^2^–MS/MS-based ceftazidime quantification.

## RESULTS

### Method Validation

#### Linearity

**FIGURE 1.**
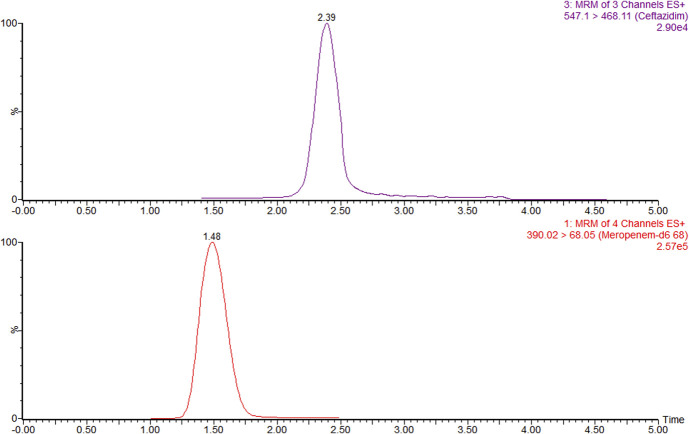
Multiple reaction monitoring chromatograms of the lower limit of quantification of the ceftazidime standard (top) and the internal standard meropenem-d_6_ (bottom). The retention time in minutes is indicated above the peak.

Least-squares linear regression was applied with a weighting factor of 1/*x*. The blank was included in the origin to ensure maximum accuracy at the lower concentrations. The following calibration curve was obtained: *y* = 0.0523*x* − 0.01146 (*r* = 0.9992 and *r*^2^ = 0.9984). The bias of the back-calculated concentrations ranged from −2.0% to 9.0%. The correlation and determination coefficients were within the linearity requirements. A chromatogram of the LLOQ sample is shown in Figure 1.

#### Accuracy

All the validation results were within the accuracy requirements. The accuracy of the results ranged from 0.3% to 5.1%. The results are summarized in Table 1.

### Intraday and Interday Precision

All the validation results were within the accuracy requirements. The intraday and interday precision results ranged from 1.4% to 9.1%. The results are summarized in Table 1.

### Stability

The stability of the autosampler and short-term and long-term stability of the samples for this method were assessed. The QC samples were found to be stable after up to 72 hours of storage in the autosampler at 5°C. After 72 hours of storage, the recovery values for the QC L, QC M, and QC H samples were 110.3%, 101.9%, and 108.3%, respectively. Short-term stability was assessed after storage for up to 1 week at −20°C. The recovery values for the QC L, QC M, and QC H samples were 86.7%, 95.1%, and 91.7%, respectively, after 48 hours of storage, and 73.0%, 80.4%, and 71.4%, respectively, after 1 week of storage. Long-term stability was assessed after storage for up to 3 months at −80°C. The recovery values for the QC L, QC M, and QC H samples were 105.3%, 99.2%, and 102.1%, respectively.

### Carryover Effect

The carryover effect was assessed by analyzing a blank sample after analysis of the highest calibration standard in duplicate. The carryover effect was 52.6%, which did not meet the requirements.

### Matrix Effects

Three different calibration curves were obtained for the 3 different matrices. The slopes of the curves are presented in Table 3.

**TABLE 3.** Results of the Matrix Effects

Matrix	Calibration Curve	*r* ^2^	*r*	Weighting Factor
MilliQ	*y* = 0.0409*x* + 0.0173	0.9990	0.9980	1/*x*
Pig vitreous humor	*y* = 0.0379*x* + 0.0017	0.9972	0.9986	1/*x*
Human vitreous humor	*y* = 0.0497*x* + 0.0042	0.9960	0.9980	1/*x*

### Clinical Application to Patient Samples

**FIGURE 2.**
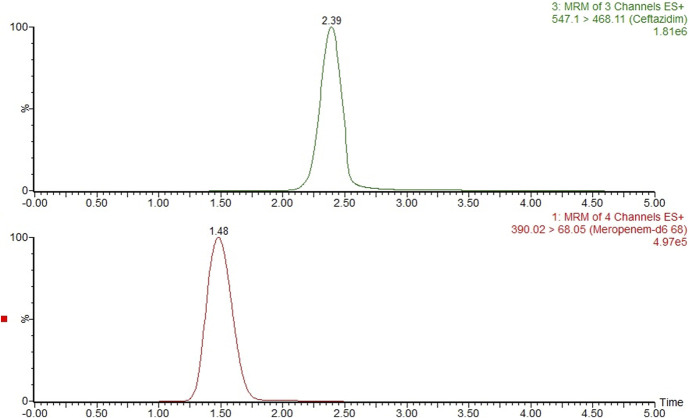
Multiple reaction monitoring chromatograms of ceftazidime in an actual clinical sample (top) and the internal standard meropenem-d_6_ (bottom)_._ The retention time in minutes is indicated above the peak.

Target-site concentrations of ceftazidime in the VH were measured in 72 patient samples. Most concentrations were within the validated range. However, 3 concentrations were below the LLOQ and 23 were above the validated ULOQ. Some samples were collected directly after ceftazidime administration, which may explain the higher concentrations. An extracted ion chromatogram of the results for a clinical sample is shown in Figure 2.

## DISCUSSION

In this study, the conformation of a UPC^2^–MS/MS method to international guidelines for the quantification of ceftazidime in the VH was validated.^8,11^

The validation results showed that the linear range of the method was from 1.4 to 99.6 mg/L. All results were within the accuracy and precision requirements. Ceftazidime was verified to remain stable in the VH for the short term up to 48 hours at −20°C, which is comparable with its reported stability at −20°C in plasma.^3,12,13^ Ceftazidime in the VH was found to be stable for up to 3 months at −80°C, which is also comparable to its stability at −80°C in plasma.^14^ Therefore, the storage of ceftazidime samples in a freezer at −80°C is recommended for periods longer than 48 hours. Furthermore, a carryover effect outside the acceptable limits was observed. However, 5 LLOQ samples that were analyzed after the blank sample containing the carryover were measured within 15% of accuracy. Because the carryover did not affect the measured LLOQ concentrations after the blank sample, it was concluded that the injection of a blank sample after each patient sample would be an appropriate solution to account for possible carryover effects. In addition, the matrix effect of human VH was determined by comparing the effects of 3 different matrices (Milli-Q water, pig VH, and human VH). The absolute areas for ceftazidime in each calibration standard curve were comparable among the matrices. The differences in the slopes among the 3 calibration curves were because of the areas of the internal standards, which differed significantly for each matrix. Therefore, human VH should be used to prepare the calibration standards and QC samples to account for matrix effects. This observation may be considered a limitation of the quantification method because blank material of the human VH is difficult to obtain. For matrices that are expensive or difficult to obtain, the feasibility of using a surrogate matrix^15^ such as animal VH or Milli-Q water to prepare calibration standards and QC samples would be of immense benefit. However, this limitation may be overcome by using an isotope-labeled internal standard of ceftazidime because the use of such internal standards in MS-based assays is an effective way to correct (potential) matrix effects.^10,16^ The validated UPC2–MS/MS method was successfully applied to 72 clinical samples. Although most concentrations were within the validated range, 3 values were below the LLOQ and 23 were above the validated range. Two additional QC concentration levels (114 and 228 mg/L, respectively, covering all measured concentrations) were prepared to evaluate the accuracy of the measured concentrations above the validated ULOQ. The measurements of these QC samples, analyzed in duplicate simultaneously with the patient samples, were within 15% of accuracy. Therefore, the accuracy of concentrations up to 228 mg/L was ensured. Hence, this method is suitable for quantifying ceftazidime in the VH.^1–16^

## CONCLUSION

In this study, a UPC^2^–MS/MS-based method for quantifying ceftazidime in the VH was successfully validated and applied to actual clinical samples. The determination of ceftazidime levels at the infection site may improve the efficacy of the drug treatment, thereby promoting the quick recovery of patients and lowering the risk of antibiotic resistance.

## References

[1] van Halsema J Jansen R Heineken A Validation of a multi-species-specific pcr panel to diagnose patients with suspected postoperative bacterial endophthalmitis. Acta Ophthalmol. 2022;100:e827–e832. 34258875 10.1111/aos.14964 PMC9291183

[2] Mishra D Gade S Glover K Vitreous humor: composition, characteristics and implication on intravitreal drug delivery. Curr Eye Res. 2023;48:208–218. 36036478 10.1080/02713683.2022.2119254

[3] Bahmany S Abdulla A Ewoldt TMJ High-throughput analysis for the simultaneous quantification of nine beta-lactam antibiotics in human plasma by UPC^2^-MS/MS: method development, validation, and clinical application. J Pharm Biomed Anal. 2022;219:114904. 35772234 10.1016/j.jpba.2022.114904

[4] Bahmany S Holst A Hoogendoorn MH Quantification of cefuroxime and flucloxacillin in synovial tissue and bone using ultra-performance convergence chromatography-tandem mass spectrometry. J Chromatogr B Analyt Technol Biomed Life Sci. 2024;1241:124169. 10.1016/j.jchromb.2024.124169 38815354

[5] Demir Z Bahmany S Bethlehem C Quantification of beta-lactam antibiotics cefuroxime and flucloxacillin in human synovial fluid, using ultra-performance convergence chromatography-tandem mass spectrometry. J Chromatogr B Analyt Technol Biomed Life Sci. 2021;1173:122696. 10.1016/j.jchromb.2021.122696 33872930

[6] Kalikova K Slechtova T Vozka J . Supercritical fluid chromatography as a tool for enantioselective separation; a review. Anal Chim Acta. 2014;821:1–33. 24703210 10.1016/j.aca.2014.02.036

[7] U.S. Food and Drug Administration. U.S. Department of Health and Human Services; 2001.

[8] U.S. Department of Health and Human Services. Bioanalytical method validation Guidance for industry. Available at: https://www.fda.gov/files/drugs/published/Bioanalytical-Method-Validation-Guidance-for-Industry.pdf. Accessed May, 2018.

[9] European Medicines Agency. Guideline on bioanalytical method validation. Available at: https://www.ema.europa.eu/en/documents/scientific-guideline/guideline-bioanalytical-method-validation_en.pdf. Accessed May, 2018 10.4155/bio.12.44 22533559

[10] Van Eeckhaut A Lanckmans K Sarre S Validation of bioanalytical LC-MS/MS assays: evaluation of matrix effects. J Chromatogr B Analyt Technol Biomed Life Sci. 2009;877:2198–2207. 10.1016/j.jchromb.2009.01.003 19179125

[11] EUCAST. Clinical breakpoints and dosing of antibiotics. Available at: https://www.eucast.org/clinical_breakpoints. Accessed January 2, 2023.

[12] Mortensen JS Jensen BP Zhang M . Preanalytical stability of piperacillin, tazobactam, meropenem, and ceftazidime in plasma and whole blood using liquid chromatography-tandem mass spectrometry. Ther Drug Monit. 2019;41:538–543. 31306394 10.1097/FTD.0000000000000650

[13] Martens-Lobenhoffer J Angermair S Bode-Boger SM. Quantification of ceftazidime/avibactam in human plasma and dried blood spots: implications on stability and sample transport. J Chromatogr B Analyt Technol Biomed Life Sci. 2022;1193:123164. 10.1016/j.jchromb.2022.123164 35196625

[14] Bahmany S Ewoldt TMJ Abdulla A . Stability of 10 beta-lactam antibiotics in human plasma at different storage conditions. Ther Drug Monit. 2023;45:606–615. 37199408 10.1097/FTD.0000000000001100 PMC10497202

[15] Wakamatsu A Ochiai S Suzuki E Proposed selection strategy of surrogate matrix to quantify endogenous substances by Japan Bioanalysis Forum DG2015–15. Bioanalysis. 2018;10:1349–1360. 30182726 10.4155/bio-2018-0105

[16] Xu RN Fan L Rieser MJ . Recent advances in high-throughput quantitative bioanalysis by LC-MS/MS. J Pharm Biomed Anal. 2007;44:342–355. 17360141 10.1016/j.jpba.2007.02.006

